# Donor NK and T Cells in the Periphery of Lung Transplant Recipients Contain High Frequencies of Killer Cell Immunoglobulin-Like Receptor-Positive Subsets

**DOI:** 10.3389/fimmu.2021.778885

**Published:** 2021-12-13

**Authors:** Anna-Maria Hitz, Kim-Alina Bläsing, Bettina Wiegmann, Ramon Bellmàs-Sanz, Evgeny Chichelnitskiy, Franziska Wandrer, Lisa-Marie Horn, Christine Neudörfl, Jana Keil, Kerstin Beushausen, Fabio Ius, Wiebke Sommer, Murat Avsar, Christian Kühn, Igor Tudorache, Jawad Salman, Thierry Siemeni, Axel Haverich, Gregor Warnecke, Christine S. Falk, Jenny F. Kühne

**Affiliations:** ^1^ Institute of Transplant Immunology, Hannover Medical School, Hannover, Germany; ^2^ Department of Cardiothoracic, Transplantation and Vascular Surgery, Hannover Medical School, Hannover, Germany; ^3^ German Center for Lung Research, DZL, BREATH Site, Hannover, Germany; ^4^ Department of Cardiac Surgery, Heidelberg University Hospital, Heidelberg, Germany; ^5^ Department of Cardiac Surgery, University Hospital of Duesseldorf, Duesseldorf, Germany; ^6^ Department of Cardiothoracic Surgery, University Hospital Jena, Jena, Germany; ^7^ German Center for Infection Research, DZIF, TTU-IICH, Hannover-Braunschweig, Germany

**Keywords:** lung transplantation, passenger leukocytes, NK cells, T cells, killer cell immunoglobulin-like receptor, primary graft dysfunction, cold ischemic time

## Abstract

**Introduction:**

For end-stage lung diseases, double lung transplantation (DLTx) is the ultimate curative treatment option. However, acute and chronic rejection and chronic dysfunction are major limitations in thoracic transplantation medicine. Thus, a better understanding of the contribution of immune responses early after DLTx is urgently needed. Passenger cells, derived from donor lungs and migrating into the recipient periphery, are comprised primarily by NK and T cells. Here, we aimed at characterizing the expression of killer cell immunoglobulin-like receptors (KIR) on donor and recipient NK and T cells in recipient blood after DLTx. Furthermore, we investigated the functional status and capacity of donor *vs*. recipient NK cells.

**Methods:**

Peripheral blood samples of 51 DLTx recipients were analyzed pre Tx and at T0, T24 and 3wk post Tx for the presence of HLA-mismatched donor NK and T cells, their KIR repertoire as well as activation status using flow cytometry.

**Results:**

Within the first 3 weeks after DLTx, donor NK and T cells were detected in all patients with a peak at T0. An increase of the KIR2DL/S1-positive subset was found within the donor NK cell repertoire. Moreover, donor NK cells showed significantly higher frequencies of KIR2DL/S1-positive cells (p<0.01) 3wk post DLTx compared to recipient NK cells. This effect was also observed in donor KIR^+^ T cells 3wk after DLTx with higher proportions of KIR2DL/S1 (p<0.05) and KIR3DL/S1 (p<0.01) positive T cells. Higher activation levels of donor NK and T cells (p<0.001) were detected compared to recipient cells *via* CD25 expression as well as a higher degranulation capacity upon activation by K562 target cells.

**Conclusion:**

Higher frequencies of donor NK and T cells expressing KIR compared to recipient NK and T cells argue for their origin in the lung as a part of a highly specialized immunocompetent compartment. Despite KIR expression, higher activation levels of donor NK and T cells in the periphery of recipients suggest their pre-activation during the *ex situ* phase. Taken together, donor NK and T cells are likely to have a regulatory effect in the balance between tolerance and rejection and, hence, graft survival after DLTx.

## Introduction

Double lung transplantation (DLTx) remains the only curative treatment option for end-stage lung diseases (1). Despite continuous progress in the optimization of the transplantation procedure, clinical outcome of patients undergoing DLTx still is poorer compared to other solid organ transplantations (2). Survival rates after DLTx are limited early due to primary graft dysfunction (PGD), occurring in the first 72 hours after transplantation. Later, survival is impaired by chronic lung allograft dysfunction (CLAD), comprising bronchiolitis obliterans syndrome (BOS) and restrictive allograft syndrome (RAS), which develop typically later than two years after DLTx. PGD grade 2 and 3 have been associated with significantly higher short-term as well as a negative effect on long-term outcome. While PGD has been extensively examined in terms of risk factors, epidemiology and treatment options (3), the underlying immunological mechanisms are still not completely understood. Since T cells represent the main effector cells of the adaptive immunity, they are the preferred subset in studies addressing allograft including lung rejection. In contrast, the role of natural killer (NK) cells in the context of solid organ transplantation is still discussed controversially as they have been shown to be involved in both graft rejection and tolerance induction in different models (4). Recently, the missing-self genetics of KIR-ligand mismatches has been discussed to contribute to microvascular rejection (5, 6). NK cells act as first defense line of the innate immune system against pathogens, capable of producing cytokines and possessing cytotoxic activity. Moreover, they have the ability to discriminate between cells of self and non-self origin, using an inhibitory recognition system of self-human leukocyte antigen (HLA) class I molecules (7, 8). The 'missing self-hypothesis' proposes that recognition of self HLA class I molecules inhibits their lytic activity, while the absence or downregulation of self HLA class I, i.e. missing self, leads to direct recognition and lysis of target cells (8, 9). The lytic activity of NK cells is thoroughly regulated by a multidirectional interaction of inhibitory and activating receptors, such as killer cell immunoglobulin-like receptors (KIR), C-type lectin and natural cytotoxicity receptors (NCR) (10). Activating KIR-S and inhibitory KIR-L genes encode surface receptors that recognize primarily two groups of HLA-C alleles and HLA-B supratypes, respectively. In addition to NK cell regulation, KIR can also be expressed by certain CD8^+^ T cell subsets and act as inhibitors of TCR signaling. Hence, both NK and T cell subsets expressing these KIR receptors may play a crucial role in the field of organ, especially lung transplantation due to its unique tissue-resident immune repertoire (11). In genetic analyses, a negative association of inhibitory KIR genes within haplotype A was shown for long-term outcome after DLTx suggesting that the KIR-ligand mismatch system may have clinical relevance for lung Tx (12).

Due to this capacity of NK and T cells for allorecognition, these regulatory mechanisms are of critical importance especially in the context of HLA mismatched settings like lung transplantation. Although the appearance of donor passenger leukocytes migrating from the implanted lung into the periphery of the recipient has been described decades ago, the respective NK cell subsets have not been characterized so far. This transient lymphocyte chimerism in the blood of lung recipients has first been described in the 1990s and especially donor NK and T cells were detected for up to four weeks after transplantation (13). The clinical impact of donor cells on lung allograft survival has been discussed ever since, with recent findings suggesting that special subsets of donor-derived T cells, i.e. tissue-resident memory T (TRM) cells, in bronchioalveolar lavage may be associated with lower incidence of primary graft dysfunction (14). Since NK and T cells were shown to account for the major passenger leukocyte populations (13) comprising important subsets of the innate and adaptive immune system, we focused on these two major effector cell populations.

In our study, 51 lung transplant recipients were analyzed for the kinetics of donor passenger NK and T cells in recipient blood directly after lung transplantation and at three weeks post Tx. We could demonstrate the existence of high frequencies of donor NK and T cells directly after transplantation in all recipients, which generated a transient lymphocyte chimerism within the first three weeks after DLTx. Unexpectedly, NK cells represented higher relative proportions of donor cells compared to T cells. In addition, the characterization of donor and recipient NK and T cell subsets revealed high frequencies of KIR-positive NK and T cell subsets, particularly with respect to KIR2DL/S1^+^ subsets. Moreover, donor NK cells displayed higher cytotoxic activity at early stages compared to recipient NK cells. Correlation analyses were performed with clinical parameters but did not show a significant impact on primary graft dysfunction. However, higher KIR^+^ NK cell frequencies were observed in lung recipients with longer cold-ischemic times, suggesting an impact of the ischemia/reperfusion injury. Taken together, this early NK and T cell chimerism may rather be involved in the long-term balance between rejection and tolerance in the lung transplant setting.

## Materials and Methods

### Patients and Sample Collection

Blood samples and clinical data of 51 patients undergoing DLTx were collected and preserved as peripheral blood mononuclear cells (PBMC). PBMC of patients were isolated using Biocoll Separating Solution (Biochrom/Merck, Darmstadt, Germany) as described. The study was approved by the ethics committee at the Hannover Medical School (no. 122-2007, 2500/2014) and all patients provided written informed consent. Venous whole blood samples were collected at the following time points: before (pre), 4-5 hours (T0), 24 hours (T24) and three weeks after DLTx (3wk). Donor and recipient demographics of the entire cohort (n=51) and the respective subgroups for the various analyses are summarized in **Table 1**. No induction therapy is applied to recipients and immunosuppression by calcineurin-inhibitors (CNI), steroids and mycophenolate mofetil (MMF) is administered intraoperatively. PGD scores were ranked according to oxygenation index and the presence of pulmonary radiographic infiltrates (15). Donor lungs were perfused with 2-3 L of Perfadex/Celsior perfusion solution and kept on cold storage in the same solution (perfusate). Perfusates of donor lungs were collected at the end of cold ischemic phase, cold ischemic times (CIT) and cross-clamp times (CCT) were documented. For collection of immune cells migrating or washed out of the allograft, perfusates were centrifuged at 453 x g for 15 min, cells were analyzed by flow cytometry and cell-free perfusate was stored at -20°C.

**Table 1:** Demographic characterization of lung transplanted patients.

Characteristics	Study cohort (n = 51)	HLA panel (n = 39)	NK/T panel (n = 33)	KIR panel (n = 14)	Degranulation assay (n = 10)
**Donor**					
Age at donation, y±SEM	47.37±2.21	48.72±2.56	45.36±2.7	51.71±4.53	40.6±5.3
sex, n (% male)	27 (53%)	20 (51%)	15 (45%)	4 (29%)	4 (40%)
**Recipient**					
Age at LTx, y±SEM	48.8±2.01	48.46±2.18	49.48±2.58	52.5±2.87	42.6±5.91
Sex, n (% male)	26 (51%)	19 (49%)	17 (52%)	6 (43%)	4 (40%)
Transplant indication, n (%)					
COPD	15 (29%)	11 (28%)	10 (30%)	5 (36%)	1 (10%)
IPF	17 (33%)	13 (33%)	11 (33%)	5 (36%)	4 (40%)
CF	10 (20%)	8 (21%)	6 (18%)	1 (7%)	3 (30%)
PIPH	3 (6%)	2 (5%)	2 (6%)	0 (0%)	1 (10%)
Others	6 (12%)	5 (13%)	4 (13%)	3 (21%)	1 (10%)
CCT, min±SEM	561.77±17.63	569.65±20.43	561.94±23.79	609.82±38.84	536.8±41.47
CIT, min±SEM	446.86±18.77	435.38±22.12	442.39±22.64	351.64±41.32*	463.2±34.54
PGD (2-3), n (%)	12 (24%)	12 (31%)	6 (18%)	4 (29%)	3 (30%)
T24	10 (20%)	10 (26%)	6 (18%)	4 (29%)	3 (30%)
T48	10 (20%)	10 (26%)	5(15%)	3 (21%)	3 (30%)
T72	3 (6%)	3 (8%)	1 (3%)	1 (7%)	1 (10%)
CLAD at end of follow-up, n (%)	9 (18%)	7 (18%)	5 (15%)	2 (14%)	2 (20%)
BOS	6 (12%)	4 (10%)	5 (15%)	2 (14%)	1 (10%)
RAS	3 (6%)	3 (8%)	0 (0%)	0 (0%)	1 (10%)
Treatment group, n (%)					
SOC	39 (76%)	27 (69%)	27 (82%)	7 (50%)	9 (90%)
EVLP	12 (24%)	12 (31%)	6 (18%)	7 (50%)	1 (10%)

BOS, bronchiolitis obliterans syndrome; CCT, cross clamp time; CF, cystic fibrosis; CIT, cold ischemic time; CLAD, chronic lung allograft dysfunction; COPD, chronic obstructive pulmonary disease; EVLP, ex-vivo lung perfusion; IPF, idiopathic pulmonary fibrosis; PGD, primary graft dysfunction; PIPH, primary idiopathic pulmonary hypertonia; RAS, restrictive allograft syndrome; SOC, standard of care; y, years.

Data are mean values ± standard error of mean (SEM). Asterisks show significant (p < 0.05) differences between HLA/BD/KIR/degranulation panels and study cohort (unpaired t-test, Mann-Whitney test).

### Detection of Donor-Derived Passenger Leukocytes in Peripheral Recipient Blood by Flow Cytometry

PBMC were thawed and incubated with live/dead yellow fluorescent reactive dye (ThermoFisher Scientific, Waltham, MA, USA), washed and stained with unconjugated donor HLA allele-specific or isotype control antibodies (**Supplementary Table 1**) for 30 min on ice, washed twice, followed by staining with secondary PB-, PE- and FITC-labeled goat-anti mouse IgG or IgM (GaM) mAb (**Supplementary Table 1**). T and NK cell subsets were identified with fluorochrome-conjugated lineage marker mAb (summarized in **Supplementary Table 1**), including mAb specific for KIR2D and 3D receptors. Of note, inhibitory and activating KIR genes cannot be distinguished by these mAb due to the identical extracellular domains of long inhibitory and short activating KIR. All staining steps were performed for 30 min at 4°C and PBMC were washed with FACS buffer (PBS plus 0.1% sodium azide, Sigma-Aldrich, Munich, Germany, 1% FBS ThermoFisher Scientific, Waltham, MA, USA). Multi-color FACS analyses were performed using LSR II flow cytometer and the Diva software (8.0.1, BD Biosciences, San Diego, CA, USA).

### Functional NK Cell Degranulation Assay

Degranulation assays were performed using recipient PBMC as previously described (16). PBMC were consecutively stained with unconjugated donor HLA allele-specific and secondary PE-labeled GaM mAb at room temperature for 30 minutes respectively, washed and incubated with FITC-labeled CD107a mAb and K562 target cells (16, 17) (1:1) for 4 h at 37°C/5% CO_2_ with addition of 50 µM monensin (Sigma-Aldrich, St. Louis, MO; USA) after the 1^st^ h. After two washing steps, cells were stained with CD56, CD16 for NK cells and CD3 as exclusion of T cells for 30 minutes at 4°C, washed twice and resuspended with FACS buffer. Cells were acquired using LSR II flow cytometer and the Diva software.

### Statistical Analysis

All statistical analyses were generated using GraphPad Prism (Version 8, San Diego, CA, USA). D’Agostino-Pearson omnibus normality test and Kolmogorov-Smirnov normality test were applied to assess data distribution. Statistical analyses (two-way ANOVA with Sidak´s multiple comparison test and Tukey´s multiple comparison test, one-sample t-test, paired t-test, Wilcoxon test and Pearson correlation) were performed as indicated in the Figure legends. P values <0.05 were considered significant.

## Results

### The Proportion of NK Cells Increases While the Frequency of T Cells Decreases in the Periphery of DLTx Recipients Directly After Transplantation

**Figure 1:**
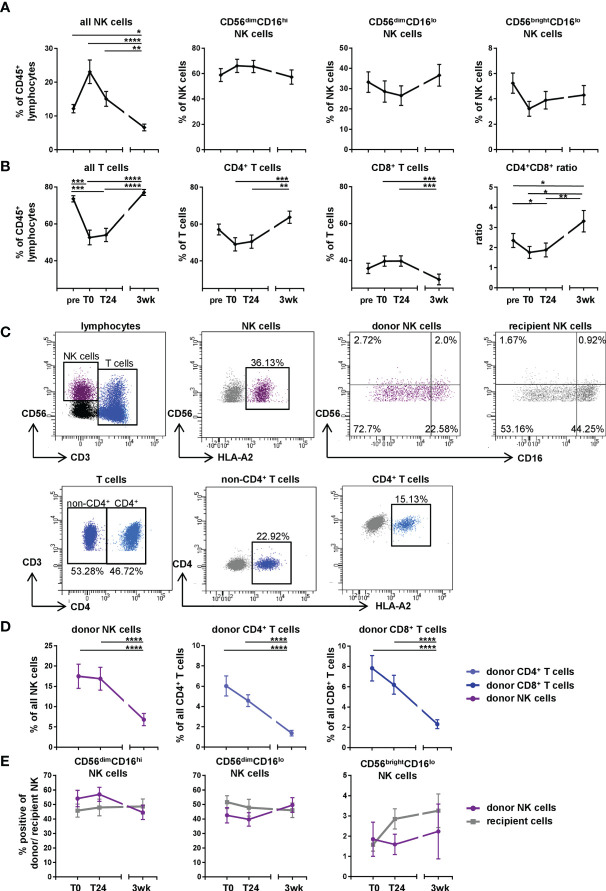
Changes in NK and T cell subsets in first three weeks after DLTx are also mediated by donor cells. **(A, B)** Frequencies of NK and T cell subsets (n = 33 of the 51 patients, **Table 1**) in peripheral blood of DLTx recipients were analyzed pre, directly post (T0), 24 hours (T24) and three weeks after transplantation (3wk). **(A)** NK cells were gated as CD45^+^CD3^-^CD56^+^ and NK cells subsets were further discriminated based on CD56 and CD16 expression. **(B)** T cells were identified as CD45^+^CD3^+^CD56^-/+^ ('all T cells'), CD3^+^CD4^+^ ('CD4^+^ T cells') and CD3^+^CD8^+^ ('CD8^+^ T cells'). **(C)** Gating strategy for donor NK and T cells in peripheral blood of one representative patient #14 directly post DLTx is shown. Donor and recipient cells were discriminated *via* HLA class I mismatch using anti-HLA-A2 specific Ab (**Supplementary Table 1**). Here, CD3^+^CD4^+^ T cell and CD3^+^non-CD4^+^ T cell subsets were defined. **(D)** Frequencies of donor NK and T cells (n = 39, **Table 1**) in peripheral blood of DLTx patients directly post, 24 hours post and three weeks after transplantation are shown. **(E)** Frequencies of donor and recipient NK cells (n = 14) regarding their CD56 and CD16 expression were analyzed. Cells were gated as described in panel **(C)** Statistical analysis: one-way ANOVA with Dunn´s multiple comparison test for **(A, B, D)** and two-way ANOVA with Sidak´s multiple comparison test and Tukey´s multiple comparison test for **(E)**. Data are shown as mean ± SEM, asterisks indicate p-values with *p < 0.05, **p < 0.01, ***p < 0.001, ****p < 0.0001.

In order to define the kinetics of lymphocyte subsets in lung transplant recipients within the first three weeks after DLTx, PBMC of DLTx patients (n=33 patients, **Table 1**) were analyzed for their NK and T cell subsets using multicolor flow cytometry (**Figures 1A, B**). Directly after DLTx (T0), a trend towards an increase in the proportion of NK cells within CD45^+^ leukocytes (p<0.12) could be observed before decreasing significantly (p<0.001) at T24 and three weeks to an even lower level compared to baseline pre Tx (pre 12.78% *vs.* 3wk 7.2%; p<0.05; **Figure 1A**). This peak at T0 resulted primarily from CD56^dim^CD16^hi^ NK cells, with a relative increase compared to stable CD56^dim^CD16^lo^ and CD56^bri^ NK subsets. In parallel, the proportion of T cells displayed a substantial decrease at T0, which, however, did not reach statistical significance (**Figure 1B**) with primarily CD4^+^ T cells disappearing post DLTx, returning to baseline levels after 3wk post DLTx. CD8^+^ T cells seemed to disappear less intensely, which resulted in transiently significant changes in the CD4^+^/CD8^+^ ratio. This dynamic change in the NK and T cell subset composition within the first 24h after DLTx indicates a regulated process in recipient blood, with differential effects on these lymphocyte subsets.

### Donor-Derived Lymphocytes Influence NK and T Cell Frequencies in the Periphery of DLTx Recipients

The existence of donor-derived 'passenger' lymphocytes migrating from the implanted lung into the periphery of the recipient has been known for a long time, although subset composition and fate of these donor cells remain rather unclear. Therefore, we investigated the kinetics of donor lymphocytes focusing on NK and T cell subsets (**Figures 1C, E**). To test our hypothesis that donor passenger lymphocytes contribute to these postoperative changes, NK and T cells were stained for donor HLA class I alleles at the same time points. Due to the HLA mismatch in DLTx, donor and recipient cells can easily be distinguished using HLA allele-specific Ab, which were available for 39 of the 51 patients (**Tables 1**, **2**). Donor and recipient NK and T cell subsets were then defined by their respective phenotype (**Figure 1C**). Directly post DLTx (T0), the highest frequencies of donor cells could be detected in general, with NK cells as predominant lymphocyte subset (**Figures 1C, D**), followed by CD8^+^ T cells. Control stainings for donor cells pre DLTx showed 0-0.8% background staining, which defined the detection limit of >1% (**Supplementary Figures A, B**). The majority of donor NK cells displayed a CD56^dim^CD16^hi^ phenotype at T0 with a downregulation of CD16 over the course of three weeks (**Figures 1C, E**). At three weeks post DLTx, all donor lymphocyte subsets decreased significantly compared to T0 (p<0.0001). Nevertheless, donor cells, primarily NK cells, were still detectable three weeks after DLTx. Thus, our results demonstrate a transient chimerism by donor NK and T cells during the first three weeks after lung transplantation in peripheral blood of recipients that contributes to the observed changes in NK and T cell frequencies.

**Table 2:** HLA mismatches of transplanted recipients and donated lungs.

Number of HLA class I mismatches							Number of HLA class II mismatches				
**0**	**1**	**2**	**3**	**4**	**5**	**6**	**0**	**1**	**2**	**3**	**4**
0	0	2	2	13	14	8	1	1	15	13	9

HLA, human leukocyte antigen.

### Higher Frequencies of KIR^+^ Subsets in Donor NK and T Cells Are Present Within the First Three Weeks After DLTx Compared to Recipient NK and T Cells

**Figure 2:**
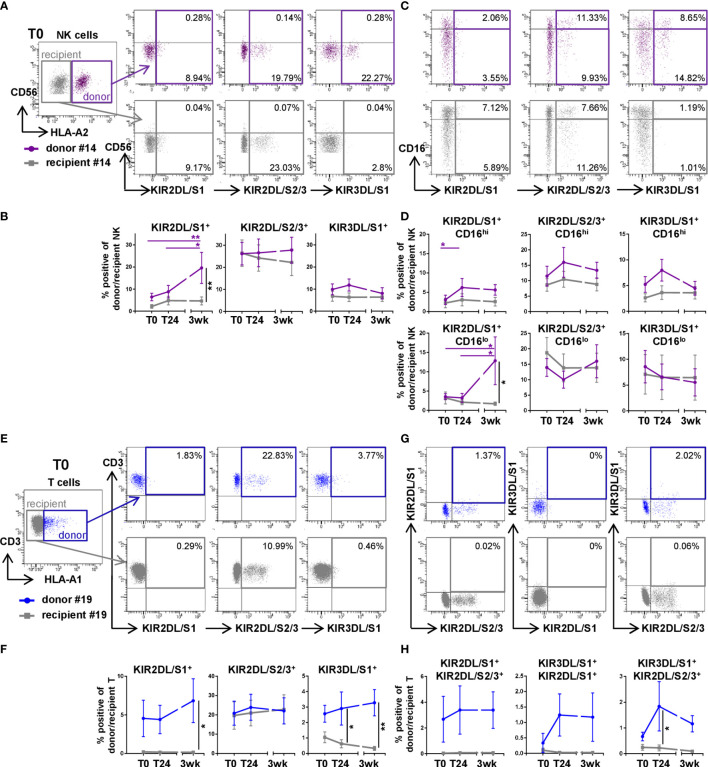
The proportion of killer cell immunoglobulin-like receptors (KIR) on donor NK and T cells is higher compared to recipient cells within the first three weeks after DLTx. **(A–D)** KIR2DL/S1, KIR2DL/S2/3 and KIR3DL/S1 surface expression on NK cells (n=14) was analyzed in peripheral blood of double-lung transplant recipients directly post (T0), 24 hours (T24) and three weeks after transplantation (3wk). Donor and recipient cells were distinguished by HLA mismatch: NK cells of the representative donor #14 were identified by HLA-A2 staining **(A, C)** T cells of the representative donor #19 were identified by HLA-A1 staining **(E, G)**, the gating strategies are shown in **Figures 1C** and **Supplementary Figure 1**. **(A)** Representative FACS plots (T0) and **(B)** frequencies of donor (purple colored squares) and recipient (grey colored squares) KIR on NK cells. **(C)** Representative FACS plots (T0) and **(D)** frequencies showing KIR expression on CD16^hi^ and CD16^lo^ NK cells. **(E–H)** The same KIR repertoire was assessed for T cells (n = 14) in peripheral blood of DLTx patients for the indicated time points. T cells of donor #19 were stained using anti-HLA-A1-specific Ab. **(E)** Representative FACS plots and **(F)** frequencies of KIR on donor (blue colored squares) and recipient (grey colored squares) T cells are displayed. **(G)** Representative FACS plots and **(H)** frequencies describing double positive KIR surface expression on T cells. Statistical analysis: two-way ANOVA with Sidak´s multiple comparison test and Tukey´s multiple comparison test. Data are shown as mean ± SEM, asterisks indicate p-values with *p < 0.05, **p < 0.01.

To better understand the composition of donor cells after DLTx, NK and T cells in recipient blood were analyzed regarding their KIR repertoire in 14 of the 51 patients (**Table 1**). NK cell activity is among other factors, regulated by the KIR expression at the clonal level. Furthermore, KIR can also be expressed by CD8^+^ T cells and influence their activity, including TCR signaling. Therefore, KIR2DL/S1, KIR2DL/S2/3 and KIR3DL/S1 expression in NK and T cells was assessed during the first three weeks after lung transplantation (**Figure 2**). Among all donor NK cells, the frequency of KIR2DL/S1 was increased significantly (p<0.05) at 3wk post DLTx compared to T0 and T24 (**Figures 2A, B**). Moreover, on donor cells, KIR2DL/S1 was mainly expressed by CD56^dim^CD16^lo^ NK cells (**Figures 2C, D**). Regarding KIR2DL/S2/3^+^ donor NK cells, a trend towards a switch from CD16^hi^ to CD16^lo^ donor NK cells could be observed within the first 3 weeks after DLTx. The frequency of KIR3DL/S1^+^ donor NK cells was stably low over time (**Figures 2B, D** and **Supplementary Figure 2A**). This differential dynamic of KIR^+^ donor NK subsets was independent from their HLA-C typing (data not shown). During three weeks after DLTx, the frequencies of KIR2DL/S1^+^ and KIR3DL/S1^+^ donor T cells were significantly higher compared to recipient T cells (both p<0.05; **Figures 2E, F** and **Supplementary Figure 2B**), while frequencies of KIR2DL/S2/3^+^ T cells showed no difference between donor and recipient T cells. Due to the individual variability, no significant changes could be seen for single KIR^+^ T cells over time. Only very few KIR double-positive donor T cells were detectable after transplantation (**Figures 2G, H**) indicating a rather single KIR^+^ subset composition. Recipient NK and T cells revealed low and stable KIR^+^ proportions during the first three weeks after DLTx, except for KIR2DL/S2/3. To uncover a potential age-related increase of KIR expression levels on NK and T cells, donor age was correlated to the KIR^+^ NK and T cells 24 hours after transplantation (T24) (**Supplementary Figure 3**). Neither a correlation between donor age nor the frequency of donor cells nor the percentage of KIR^+^ donor NK and T cells could be found, indicating no influence of donor age on the KIR repertoire on donor NK and T cells. Therefore, our results demonstrate a chimerism between donor and recipient NK and T cells in recipient blood during the first three weeks after DLTx with a substantial contribution of donor KIR^+^ NK cell subsets and a minor fraction of donor KIR^+^ T cells.

### Increased Activity and Functional Capacity of Donor NK and T Cells in the Periphery of the Recipient During the First Three Weeks Post DLTx

**Figure 3:**
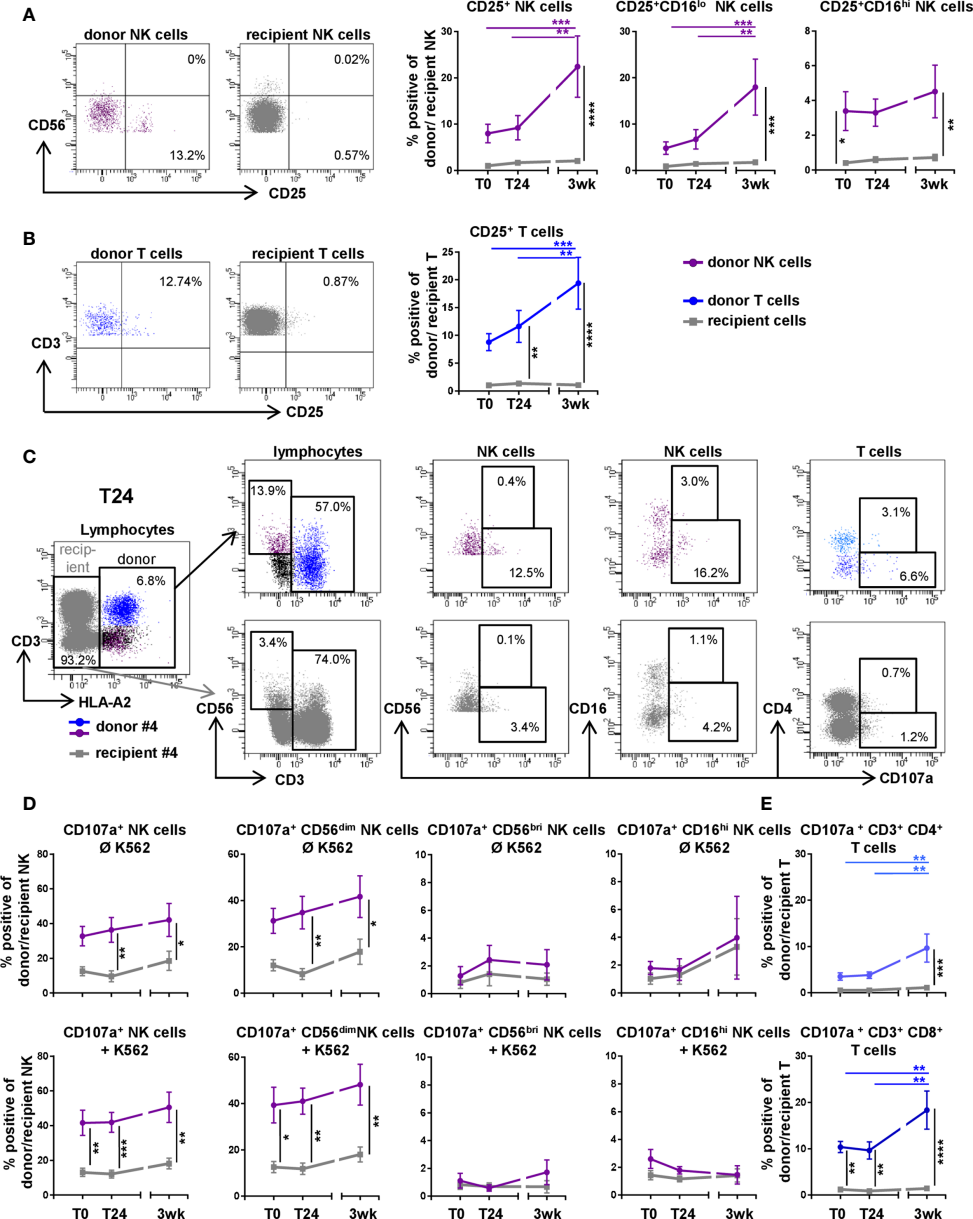
Donor NK and T cells show a higher activation level and degranulation capability three weeks after double-lung transplantation (DLTx) in comparison to recipient cells. **(A, B)** CD25 surface expression was analyzed on NK (n = 14) and T cells (n = 14) in peripheral blood of lung transplant recipients post (T0), 24 hours post (T24) and three weeks after transplantation (3wk). Underlying gating strategy is displayed in **Figure 2**. Representative FACS plots (3wk) and frequencies of **(A)** NK cells and **(B)** T cells are shown. **(C–E)** CD107a surface expression was investigated on donor and recipient NK (n = 10) and T cells (n = 10) at indicated time points with and without exposure to K562, respectively. **(C)** Gating strategy for analysis of CD107a^+^ NK and T cells in peripheral blood at T24 without K562 is shown. Events were gated on singlets. Lymphocytes were defined through FSC/SSC. Donor and recipient cells were discriminated *via* mismatch for HLA-A2. Cells were gated on CD3^-^CD56^dim/bri^ for NK cells and CD3^+^CD56^-^ for T cells. **(D)** Frequencies of CD107-expressing donor and recipient NK as well as NK cell subsets with and without exposure to K562 and **(E)** CD107-expressing donor and recipient T cells without contact to K562. Statistical analysis: two-way ANOVA with Sidak´s multiple comparison test and Tukey´s multiple comparison test. Data are shown as mean ± SEM, asterisks indicate p-values with *p < 0.05, **p < 0.01, ***p < 0.001, ****p < 0.0001.

To assess activation levels of donor NK and T cells in comparison to recipient cells in peripheral blood, we analyzed the surface expression of the activation marker CD25, IL-2 receptor alpha chain, pre, directly, 24 hours and three weeks post DLTx by flow cytometry (**Figure 3**). Over time, the proportion of CD25^+^ donor NK and T cells increased up to 20% (p<0.001), whereas recipient CD25^+^ NK and T cells remained stable at a rather low level of approximately 1% (p<0.0001 for donor *vs.* recipient at 3wk post DLTx) (**Figures 3A, B**). Three weeks post DLTx, activated donor NK cells were characterized as primarily CD56^dim^ (data not shown) CD16^lo^ NK cells (**Figure 3A**). We further analyzed donor and recipient NK cells for their functional capability, i.e. degranulation, by measuring surface expression of CD107a upon exposure to HLA-deficient target cells (K562). Comparing donor and recipient, significantly higher levels of CD107a surface expression could be detected in donor NK cells, already in the absence of K562 target cells (**Figures 3C, D** upper panel). In the presence of K562 target cells, donor NK cells displayed higher degranulation compared to recipient NK cells at all time points (all p < 0.5; **Figures 3C, D** lower panel). However, during the first 3 weeks after DLTx a trend towards increased degranulation capacity could be observed for both donor and recipient cells. Further characterization of NK cell subsets revealed that the main proportion of degranulating donor NK cells displayed a CD56^dim^CD16^lo^ phenotype. Exposure to K562 showed an additional effect neither on recipient nor on donor NK cell degranulation at any time point indicating a suppressive effect *via* immunosuppression also for donor NK cells. Gating on CD16^bri^ NK cells showed a rather poor degranulation potential, as expected (16). Regarding the degranulation capacity of T cells, again donor CD4^+^ and CD8^+^ T cells displayed significantly higher proportions of CD107a^+^ cells over the course of three weeks without K562 (p<0.01 compared to T0). CD8^+^ donor T cells also showed overall higher levels of degranulation compared to CD8^+^ T cells of the recipient (p< 0.0001 at 3wks post DLTx; **Figure 3E**). Since K562 does not stimulate T cells, these data are not shown. The presented results indicate that donor NK and T cells, predominantly CD56^dim^CD16^lo^ NK and CD8^+^ T cells, reached *per se* an activated and functional state in the periphery of the recipient, expressing higher levels of activation markers compared to the recipients own NK and T cell repertoire.

### Predominantly KIR2DL/S1- and KIR2DL/S2/3-Positive T Cells and CD56^dim^CD16^lo^ NK Cells From the Lung Allograft Are Found in the Recipient Periphery Early Post DLTx

**Figure 4:**
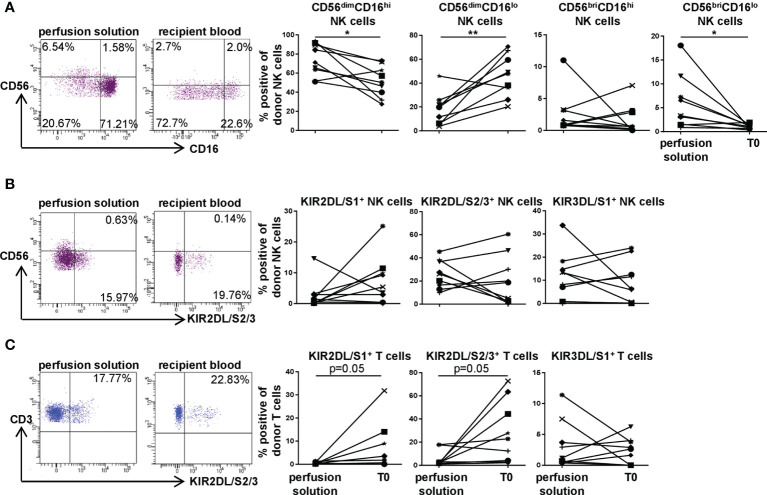
KIR repertoire on donor NK and T cells in perfusion solution distinguishes from the one in recipient blood directly post DLTx (T0). KIR2DL/S1, KIR2DL/S2/3 and KIR3DL/S1 surface expression by donor NK and T in perfusion solution and recipient blood of 9 DLTx patients was determined. Cells were gated for CD56^dim^ and CD56^bri^ NK cells as described in **Figure 1C** and for KIR as illustrated in **Figures 2A, E**. **(A)** Representative FACS plots and frequencies of donor CD56^dim^ or CD56^bri^ and CD16^hi^ or CD16^lo^ NK cells in perfusion solution and recipient blood are shown. **(B)** Representative FACS plots of KIR2DL/S2/3 and frequencies of KIR2DL/S1, KIR2DL/S2/3 and KIR3DL/S1 surface expression on donor NK cells are demonstrated. **(C)** Representative FACS plots of KIR2DL/S1 and frequencies of KIR2DL/S1, KIR2DL/S2/3 and KIR3DL/S1 expression on donor T cells. Statistical analysis: paired t-test was calculated for normally distributed data, otherwise Wilcoxon test. Asterisks indicate p-values with *p < 0.05, **p < 0.01.

We next compared donor NK and T cell subsets for their phenotypes including KIR expression in recipient blood *vs.* perfusion solution, i.e. the storage solution of the lung during the *ex situ* phase (**Figure 4**). In perfusion solution, the major NK cell phenotype was CD56^dim^CD16^hi^, while donor NK cells in recipient blood at T0 demonstrated a CD56^dim^CD16^lo^ phenotype indicating previous activation. The frequency of CD56^bri^CD16^lo^ NK cells was significantly higher (p<0.05) in perfusion solution compared to recipient periphery (**Figure 4A**). Comparing the KIR repertoire on donor NK cells in perfusion solution with recipient blood, high frequencies were observed without significant differences except for KIR2DL/S1with higher proportions on donor cells in recipient blood (**Figure 4B**). In donor T cells, higher KIR2DL/S1 and KIR2DL/S2/3 (both p=0.05) surface expression in recipient blood compared to perfusion solution was detectable (**Figure 4C**). To reveal a potential age-related influence on the KIR repertoire of NK and T cells in the perfusion solution, we correlated donor age to KIR^+^ donor NK and T cells in perfusion solution. Our results could not reveal an impact of donor age on the KIR repertoire of donor NK and T cells in perfusion solution (**Supplementary Figure 3**). Our data suggest that predominantly CD56^dim^CD16^hi^ NK cells leave the donated lung during preservation into the perfusion solution, whereas CD56^dim^CD16^lo^ NK cells may migrate primarily into recipient blood directly after DLTx. KIR expression on donor T cells was higher in the recipient blood compared to perfusion solution while this did not affect KIR^+^ subsets substantially.

### The KIR Repertoire on Donor NK and T Cells in DLTx Recipient Blood Does Not Correlate With Primary Graft Dysfunction

**Figure 5:**
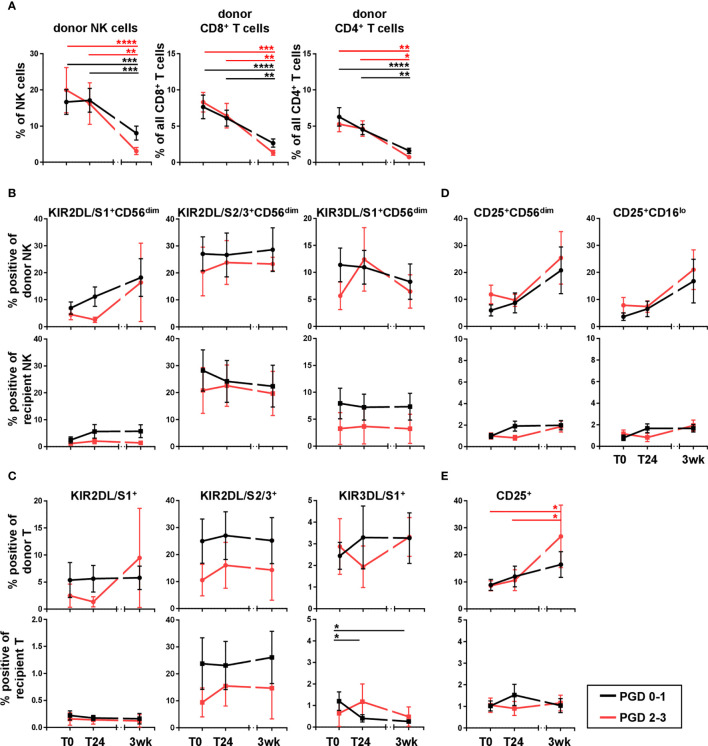
KIR repertoire of donor and recipient NK and T cells does not influence primary graft dysfunction (PGD). **(B, D)** KIR2DL/S1, KIR2DL/S2/3, KIR3DL/S1 and **(C, E)** CD25 expression on NK and T cells was analyzed in peripheral blood of lung transplant recipients post (T0), 24 hours (T24) and three weeks after transplantation (3wk). Donor and recipient cells were distinguished by HLA mismatch using anti-HLA-A2/A1 specific Ab. Cells were gated as described in **Figure 1C**. DLTx recipients were divided into two groups, PGD0-1 [n = 29 for **(A)**; n = 10 for **(B–E)**] and PGD2-3 [n = 10 for **(A)**; n = 4 for **(B–E)**], concerning their severity of PGD at 24 hours post DLTx. **(A)** Frequencies of donor NK and donor T cell subsets are shown. **(B, D)** Illustration of frequencies of KIR positive NK and T cells and **(C, E)** their CD25 expression. Statistical analysis: two-way ANOVA with Sidak´s multiple comparison test and Tukey´s multiple comparison test. Data are shown as mean ± SEM, asterisks indicate p-values with *p < 0.05, **p < 0.01, ***p < 0.001, ****p < 0.0001.

Next, we wanted to analyze the clinical impact of donor NK and T cells in the recipient periphery and their KIR repertoire on primary graft dysfunction (PGD), a major cause of early graft failure and poor transplant outcome. Therefore, lung transplant recipients were divided into two groups according to their PGD scores. PGD was assessed 24 hours post DLTx and graded in PGD 0-1 (no and low degree of severity) and PGD 2-3 (high degree of severity). Despite a very small sample size, both groups were compared with respect to their donor NK, T cell frequencies, and the KIR repertoire on donor and recipient cells. In general, no differences could be detected for the frequencies of donor NK and CD4^+^ or CD8^+^ donor T cells between both PGD groups (**Figure 5A**). Furthermore, the data revealed no significant distinction between PGD0-1 and PGD2-3 focusing on KIR repertoire on donor and recipient NK and T cells, although the frequency of KIR^+^ NK and T cells was slightly lower in DLTx patients suffering PGD2-3 (**Figures 5B, C**). The association of PGD and the activation marker CD25 was also analyzed. No differences in the CD25 surface expression on donor NK cells comparing PGD 0-1 and PGD2-3 could be shown (**Figure 5D**). In contrast, an increase in the frequency of CD25^+^ donor T cells (p<0.05) in patients with PGD2-3 over time could be demonstrated (**Figure 5E**). The proportion of CD25^+^ donor T cells in PGD-01 patients also slightly increased without reaching statistical significance. The difference in CD25^+^ donor T cells between PGD2-3 and PGD0-1 patients at three weeks was also not significant. Taken together, our results indicate that neither frequency nor KIR repertoire of donor NK and T cells did affect the early clinical outcome, i.e. PDG, after lung transplantation.

### Frequencies of KIR^+^ Donor NK Cells Directly After DLTx Tend to Increase With Longer Cold Ischemic Times (CIT)

**Figure 6:**
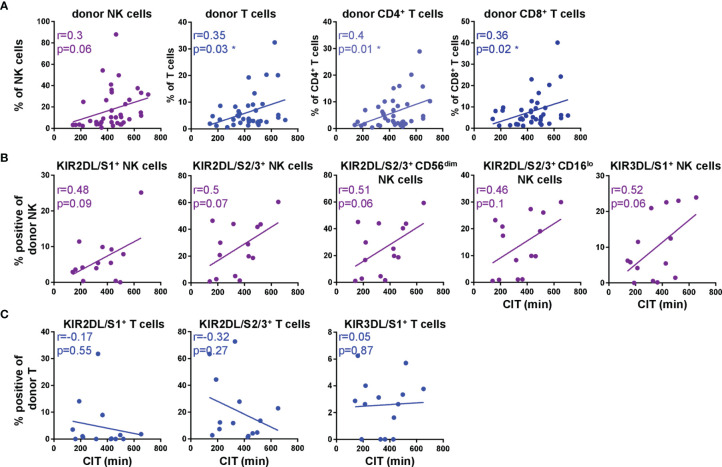
KIR^+^ donor NK cell subsets increase with longer cold ischemic time (CIT). **(A)** Scatter plots show linear regression of CIT to donor NK and T cell subsets (n = 39) and **(B)** KIR surface expression on donor NK cells (n = 14) and **(C)** T cells (n = 14). Each dot represents one patient. Gating strategy for donor NK and T cells is shown in **Figure 1C** and for KIR in, **(Figures 2A, E)** Statistical analysis: linear regression and correlation analysis by Pearson correlation. Asterisks indicate p-values with *p < 0.05.

Little is known about the impact of the cold static preservation on the explanted lungs in terms of NK and T cell kinetics in recipient blood and clinical outcome. Therefore, we correlated cold ischemic times (CIT) with frequencies of donor NK and T cells at T0 (**Figure 6**). Our data mirrored a positive correlation between the duration of CIT and frequencies of donor NK as well as CD4^+^ and CD8^+^ T cell subsets in recipient blood directly post DLTx (**Figure 6A**). Especially the proportion of CD4^+^ donor T cells increased with longer CIT (r=0.4; p=0.01). Regarding the influence of CIT on the KIR repertoire of donor NK cells at T0 (**Figure 6B**), a trend for a correlation was found between CIT and all analyzed KIR on donor NK cells (KIR2DL/S1^+^ r=0.48, p=0.09; KIR2DL/S2/3^+^ r=0.5; p=0.07 and KIR3DL/S1^+^ r=0.52; p=0.06). Particularly the KIR2DL/S2/3^+^CD56^+^ NK cells showed a positive correlation to CIT. In contrast, we could not show any correlation between CIT and the KIR repertoire on donor T cells (**Figure 6C**, **Supplementary Figure 4**). In summary, these results imply that both donor NK and T cells generally are influenced by CIT. Furthermore CIT has an impact on KIR^+^ NK cell subsets whereas the duration of the *ex situ* time of the lung has no influence on the KIR repertoire on donor T cells. These results may have a so far underestimated impact on the donor/recipient distribution in lung transplantation.

## Discussion

Recently, the impact of NK cells and the genetics associated with the KIR ligand mismatch has been shown for kidney transplantation (5), which raises also the question for other organs, especially those with tissue-resident cells like the lung. Passenger lymphocytes can be found in the peripheral blood of lung transplant recipients (13, 18), but the knowledge on their kinetics, phenotype and relevance in the lung transplant setting is still rather scarce. Here, we aimed to elucidate donor NK and T cell characteristics in recipient blood during the first three weeks after lung transplantation with particular focus on their KIR repertoire and their impact on clinical outcome. To the best of our knowledge, these dynamics within the first three weeks after DLTx have not been studied in detail before. In the current study, we unexpectedly observed substantial changes in the composition of lymphocyte subsets in the recipient periphery immediately after DLTx. In general, our analyses revealed higher frequencies of NK cells, dominated by CD56^dim^CD16^hi^ NK cells at T0, and a lower proportion of T cells, primarily CD4^+^ T cells, directly post DLTx compared to baseline pre DLTx. Furthermore, a significant decrease in the CD4^+^/CD8^+^ T cell ratio directly post DLTx was detected. In order to identify a potential link between donor passenger cells and the dynamics in lymphocyte subsets, we focused our analyses on the first three weeks, especially the first 24 hours. This time frame was also supported by previous studies detecting donor passenger leukocytes in recipient peripheral blood during the first four weeks following lung transplantation (14).

With the HLA-based discrimination between donor and recipient leukocytes, we could proof that a transient lymphocyte chimerism, primarily by NK and T cells but not B and myeloid cells (data not shown) is involved in the dynamic changes in lymphocyte subsets after DLTx. Within the NK and T cell subsets, donor NK cells represented the highest proportion at all three time points, followed by CD8^+^ and CD4^+^ T cells. The frequency of donor CD8^+^ T cells among all CD8^+^ T cells was elevated compared to CD4^+^ T cells and, therefore, can partially explain the generally decreased CD4^+^/CD8^+^ T cell ratio directly post DLTx, which was also mediated by substantially decreased recipient CD4^+^ T cells. The reduced frequency of all T cells and the simultaneously increased proportion of NK cells directly post DLTx cannot be explained only by the augmented frequency of donor NK cells leaving the transplanted lung into the recipient periphery. Hence, it is conceivable that T cells leave the periphery, and may potentially migrate into the transplanted lung, as an immune response to the transplanted organ. This scenario has been proposed for NK cells (19). Alternatively, T cells may migrate to the lymphatic system, i.e. lymph nodes and spleen, which can also be observed during infection. Bidirectional movements of donor and recipient T and NK cells between peripheral blood and the lung allograft are capable to evoke profound changes in leukocyte subsets directly post DLTx.

Next, we aimed for a more detailed characterization of the phenotype of donor NK cells with respect to the major peripheral subsets, i.e. CD56^dim^CD16^hi^ and CD56^bri^CD16^neg^ NK cells. We focused on KIR expression, since activating and inhibitory KIR are major surface receptors regulating NK and T cell activity and consequently, might play a crucial role in the context of lung transplantation (11). To get further insights into the KIR expressing donor NK and T cell subsets, we analyzed the surface expression of the most relevant KIR on donor as well as recipient NK and T cells after DLTx. We found higher proportions of KIR2DL/S1, KIR2DL/S2/3 and KIR3DL/S1 expressing subsets in donor NK and T cells compared to recipient cells. Our results furthermore demonstrated a significant increase in frequency of KIR2DL/S1^+^ donor NK cells during the first three weeks post DLTx, whereas the frequency of KIR2DL/S1^+^ recipient NK cells remained stable at low levels over time. In addition, we studied the functionality of these donor NK and T cells in recipient blood and found donor NK cells to exhibit a higher degranulation capability compared to recipient NK cells. The poor capability of both donor and recipient NK cells to respond to HLA class I-deficient K562 cells in terms of vitro degranulation is likely caused by the onset of immunosuppression in the patient intraoperatively (20, 21). In addition, KIR surface expression increases with progressing maturation of NK and T cells (22, 23). Both aspects, enhanced degranulation capability and increased KIR expression on donor cells, suggests donor NK and T cells to have a more functional phenotype compared to recipient cells after DLTx.

In order to investigate the potential origin of donor NK cells in more detail, we also used perfusion solutions to identify the donor NK cell composition and to compare it to the passenger cells in the recipient periphery directly after DLTx (data not shown). NK cells in perfusion solutions were predominantly composed of CD56^dim^CD16^hi^ NK cells. Since donor NK cells in recipient blood were mainly represented by CD56^dim^CD16^lo^ NK cells, this change in CD16 surface expression argues for at least some degree of activation in this CD56^dim^ NK cells subset. This CD56^dim^ NK subset has also been identified as lung-resident subset in human lung parenchyma by Marquardt et al. (24). Since we primarily detected the CD56^dim^CD16^hi^ NK cell subset in perfusion solution, we propose that this subset leaves the donor lung early during preservation, whereas directly after DLTx, the CD56^dim^CD16^lo^ NK cell subset is migrating out of transplanted lung into the recipient periphery. In addition, the low CD16 expression can be an indicator for a partial activation, which may also explain their spontaneously high CD107a expression level even in the absence of K562 target cells.

Our group has previously demonstrated that downregulation of CD16 surface expression on NK cells in patients after kidney transplantation was associated with activation and induction of interferon-γ (20). Here, we detected a downregulation of CD16 surface expression on NK cells up to three weeks after DLTx, thereby showing a similar CD16 modulation also in lung transplantation. In the DLTx cohort, CD16 downregulation was even more pronounced in donor compared to recipient NK cells. In parallel, the activation marker CD25 was significantly higher expressed on both donor NK and T cells, which argues for a higher activation level in donor *vs*. recipient cells. This higher activation status of donor NK and T cells was observed continuously within the first three weeks after DLTx and may be explained by a permanent recognition of the recipient, which may be driven by the HLA mismatch, especially in KIR ligand mismatch constellations between donor and recipient, according to the ‘missing self’ hypothesis. This is rather likely to be the case since in our cohort, approximately 90% of recipients show more than three HLA class I and three HLA class II mismatches to the respective donor HLA alleles. In conclusion, we characterized donor NK and T cells to exhibit a more activated and functional phenotype compared to recipient cells in peripheral blood of lung recipients during the first three weeks following DLTx. Thus, future studies are required to further elucidate whether activated NK cells may be associated with rejection also in lung transplantation as it was shown for CD69^+^CD56^dim^ NK cells in AMR biopsies of kidney transplant recipients (25).

The clinical outcome after DLTx is still limited, predominantly due to early, i.e. PGD, and late chronic allograft dysfunction (CLAD). Since the impact of a transient chimerism has not been addressed clinically in DLTx, we investigated the relevance of donor NK and T cells in terms of PGD and CIT. In BAL of lung recipients, it has been recently shown that certain proportions of donor-derived T cells with a tissue-resident memory phenotype are associated with a lower incidence of primary graft dysfunction (14). In contrast to BAL, donor T cells were not detectable in recipient blood in these analyses, which started 4 weeks after transplantation and, hence, did not cover the very early phase directly after DLTx. Therefore, we can assume that the chimerism detected in our setting is likely to be transient and may wean after several weeks, which is also the case by own observations (data not shown).

To elucidate the function and relevance of the KIR family in the lung transplant setting, we compared the KIR repertoires on donor *vs*. recipient NK and T cells and correlated these to PGD severity, representing a major complication early after DLTx, as well as CIT. In this small cohort, we could not show a correlation between the proportions of KIR^+^ donor NK or T cells and PGD, since patients suffering from PGD2-3 did not display higher KIR frequencies. Hence, the KIR NK and T cell repertoire does not seem to have a direct effect on clinical outcome, i.e. PGD during the first 72 hours after DLTx. However, the preservation period may impinge on the frequency of donor NK cells since we observed a positive correlation between CIT and KIR^+^ NK subsets. Interestingly, the frequencies of KIR-expressing donor T cells did not increase with longer CIT, further underlining the important role of NK cells. This unexpected finding points towards a physiological impact of ischemia reperfusion injury on the mobilization of NK and T cells in lung transplantation, which is likely influenced by the inflammatory milieu of the lung during the *ex situ* phase. In addition to the known ischemic mechanisms (26), extended preservation times may also be critical for the transient chimerism and, rather indirectly, maybe also for early clinical outcome. Our findings are also supporting a previous study demonstrating a crucial role of NK cells on clinical outcome within the early phase after kidney transplantation (27). However, the mechanisms may be unrelated since in kidney transplantation, no passenger cells have been identified (28).

Acute cellular rejection (ACR), driven mainly by alloreactive cytotoxic T cells, can lead to acute graft failure, and with regards to the long-term outcome, can also result in CLAD development. Furthermore, besides NK cells, inhibitory KIRs are expressed by CD8^+^ T cells and, therefore, regulate their functions and survival in i.e. viral infections (22, 29, 30). In our DLTx cohort, we detected a significantly higher KIR3DL/S1 expression on donor compared to recipient T cells. It will be interesting to validate these findings in a larger patient cohort and to relate them to the long-term-survival of the allograft. Still, iKIR are not able to downregulate all aspects of T cell activation, as demonstrated by elegant experiments in a transgenic mouse system (31). Therefore, inhibitory KIR may be important for the fine-tuning and adjustment of T cell functions. In addition, it has been shown that NK and T cells share many functional and phenotypical properties (32). Thus, it might be possible to transfer – at least in part – conclusions for donor NK cells onto donor T cells. Yet, future studies will be needed to further elucidate the functional consequences of iKIR expression by (donor) CD8^+^ T cells in the context of lung transplantation with a focus on ACR development.

Based on the known impact of age on NK and T cell subset distributions, we correlated the KIR^+^ subsets to donor age with the aim to show whether, increasing KIR proportions may be elevated with progressing age indicating enhanced maturation, as many groups have demonstrated (22, 23). Surprisingly, no correlations between KIR expression and donor age were found indicating that the donor age only has a weak effect on the KIR repertoire on donor NK and T cells. Furthermore, due to limitations of donor lung availability these results imply that even older people could serve as potential donors in the lung transplant setting.

In conclusion, our findings reveal donor NK and T cells in the periphery of lung transplant recipients as highly activated and functional subsets and therefore might be crucial in the lung transplant setting. Further studies investigating regulatory receptors on donor NK and T cells are necessary to better understand their contribution to graft tolerance thereby improving patient survival after lung transplantation.

### Limitations of the Study

The results presented here are derived from a single transplant center with a limited sample size. Thus, expanding the patient number is desirable to substantiate our findings. Due to the incomplete HLA-C typing, we were unable to define the HLA-C KIR ligand mismatches in detail. Moreover, based on the limited availability of allele-specific α-HLA monoclonal antibodies, only selected patient combinations with donor-specific HLA class I alleles could be analyzed for the discrimination of donor *vs*. recipient cells. Moreover, it would be interesting to identify the KIR-expressing NK and T cells subsets in BAL samples representing airway compartment. The clinical correlations were limited to the early events like PGD and CIT but in the future, we plan to investigate also a possible impact on intermediate outcome like acute rejection as well as long-term outcome, i.e. CLAD. These aspects are part of ongoing studies.

## Data Availability Statement

The raw data supporting the conclusions of this article will be made available by the authors, without undue reservation.

## Ethics Statement

The studies involving human participants were reviewed and approved by ethics committee at the Hannover Medical School (no. 122-2007, 2500/2014). The patients/participants provided their written informed consent to participate in this study.

## Author Contributions

AMH and KAB performed experiments, analyzed data and wrote the manuscript. BW coordinated sample collection and contributed to writing of the manuscript. RBS and FW helped with statistics and writing of the manuscript. EC revised statistics, figures, tables and text of the manuscript. JK, KB, LMH and CN performed and supported key experiments. FI, WS, MA, CK, IT, JS, TS, AH and GW performed DLTx and were instrumental for sample and clinical data collection. CSF and JFK supervised the work, designed experiments, analyzed data and wrote the manuscript.

## Conflict of Interest

The authors declare that the research was conducted in the absence of any commercial or financial relationships that could be construed as a potential conflict of interest.

## Publisher’s Note

All claims expressed in this article are solely those of the authors and do not necessarily represent those of their affiliated organizations, or those of the publisher, the editors and the reviewers. Any product that may be evaluated in this article, or claim that may be made by its manufacturer, is not guaranteed or endorsed by the publisher.

## Supplementary Material

Supplementary Figure 1 Flow cytometry gating strategy for the discrimination of donor *vs.* recipient cells, exemplary shown for an HLA-A2-positive donor (lung) transferred into an HLA-A2-negative recipient. **(A)** Cell doublets were excluded based on the area/height ratio. In the next step, dead cells were excluded based on the life/dead staining. CD45^+^ cells were then gated and finally lymphocytes were gated based on their CD45 expression and granularity (SSC). Subsequently, CD56^+^CD3^-^ NK cells as well as CD3^+^CD56^+/-^ T cells were defined. NK cells were further discriminated into either donor or recipient origin based on HLA-A2 expression. In this example, the donor is HLA-A2^+^ and donor NK cells (CD56^+^HLA-A2^+^) are displayed in pink, whereas the recipient is HLA-A2^-^ and recipient NK cells (CD56^+^HLA-A2^-^) are shown in purple. T cells were further divided into CD3^+^CD4^+^ T cells and CD3^+^non-CD4^+^ (“non-CD4^+^”) T cells. Finally, donor as well as recipient origin for both CD4^+^ and non-CD4^+^ T cells was determined based on HLA-A2 expression (donor T cells HLA-A2^+^, recipient T cells HLA-A2^-^) and are displayed in different blue shades. **(B)** High proportions of donor NK and T cells can be detected in recipient blood directly post DLTx. Frequencies of donor NK and T cell subsets in peripheral blood of DLTx recipients were analyzed pre, directly post (T0), 24 hours (T24) and three weeks after transplantation (3wk). Donor and recipient cells were discriminated *via* HLA mismatch using anti-HLA-A2 specific Ab. Gating strategy is shown in **Figure 1C**. Statistical analysis: one-way ANOVA with Dunn´s multiple comparison test, (n = 39). Data are shown as mean ± SEM, asterisks indicate p-values with ****p < 0.0001.

Supplementary Figure 2 Exemplary flow cytometric analyses for KIR2DL/S1, KIR2DL/S2/3 and KIR3DL/S1 surface expression on donor NK and T cells in peripheral blood of double-lung transplant recipients directly post (T0), 24 hours (T24) and three weeks after transplantation (3wk). Donor and recipient cells were distinguished by HLA mismatch: NK cells of the representative donor #14 were identified by HLA-A2 staining **(A)**; T cells of the representative donor #19 were identified by HLA-A1 staining **(B)**, the gating strategies are shown in **Figure 1C** and **S1**. **(A)** Representative FACS plots of one patient of donor#14 (HLA-A2^+^; purple) and recipient#14 (HLA-A2^-^; grey) KIR on NK cells directly after DLTx (T0), 24 hours (T24) and 3 weeks following DLTx. **(B)** Representative FACS plots of one patient of donor#19 (HLA-A1^+^; blue) and recipient#19 (HLA-A1^-^; grey) KIR on T cells.

Supplementary Figure 3 Donor age might have no impact on the KIR repertoire on donor NK and T cells directly post DLTx (T0). Linear regression of donor age to **(A)** donor NK and T cell subsets (n = 39) directly post DLTx (T0) and to KIR on **(B)** donor NK cells (n = 14) and **(C)** donor T cells (n = 14) directly post DLTx (T0) is shown. Each dot represents one patient. Gating strategy for donor NK and T cells is shown in **Figure 1C** and for KIR in **Figures 2A, E**. Statistical analysis: linear regression and correlation analysis by Pearson correlation.

Supplementary Figure 4 Donor age may has no impact on the KIR repertoire on donor NK and T cells in perfusion solution. Linear regression of donor age to KIR on **(A)** donor NK cells (n = 9) and **(B)** donor T cells (n = 9) in perfusion solution is illustrated. Gating strategy for KIR in **Figures 2A, E**. Each dot represents one patient. Statistical analysis: linear regression and correlation analysis by Pearson correlation.

Supplementary Table 1 Antibodies for flow cytometry. APC, allophycocyanine; BV, brilliant violet; ECD, energy coupled dye (phycoerythrin-texas red conjugate); FITC, fluorescein isothiocyanate; PB, pacific blue; PE, phycoerythrin; PerCP, peridinin-chlorophyll-protein complex.

## Abbreviations

ACR, acute cellular rejection; BOS, bronchiolitis obliterans syndrome; CIT, cold ischemic time; CLAD, chronic lung allograft dysfunction; DC, dendritic cells; DLTx; double-lung transplantation; HLA, human leukocyte antigen; KIR, killer cell immunoglobulin-like receptor; mAb, monoclonal antibody; NK cells, natural killer cells; PBMC, peripheral blood mononuclear cells; PBS, phosphate-buffered saline; PGD, primary graft dysfunction; RAS, restrictive allograft syndrome; SOC, standard of care.
