# Supplementary figures and images for: Donor NK and T Cells in the Periphery of Lung Transplant Recipients Contain High Frequencies of Killer Cell Immunoglobulin-Like Receptor-Positive Subsets
Source: Front Immunol. 2021 Dec 13;12:778885. doi: 10.3389/fimmu.2021.778885 (PMC8710687; doi:10.3389/fimmu.2021.778885)

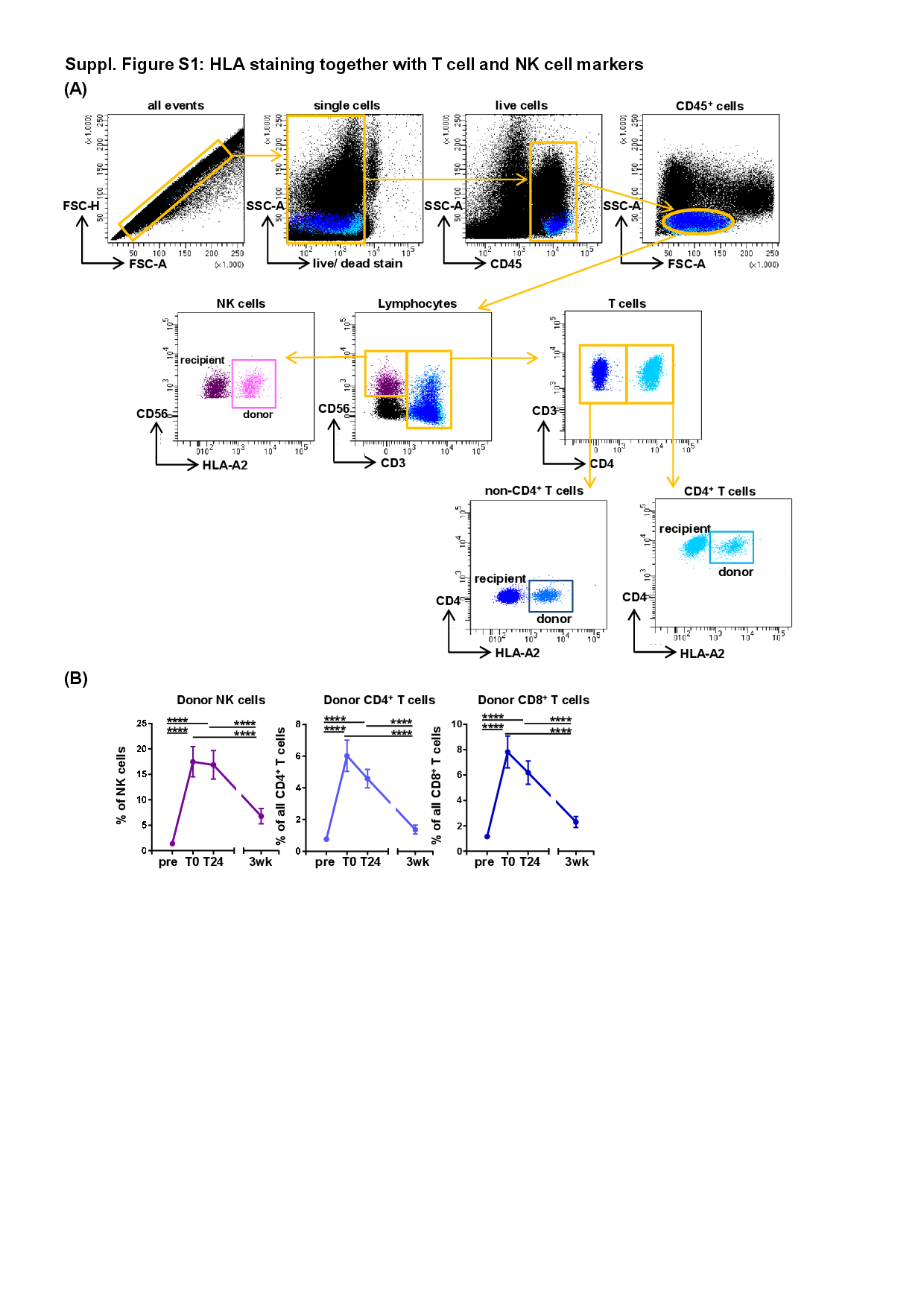

Supplement: Supplementary Figure 1 — Flow cytometry gating strategy for the discrimination of donor vs. recipient cells, exemplary shown for an HLA-A2-positive donor (lung) transferred into an HLA-A2-negative recipient. (A) Cell doublets were excluded based on the area/height ratio. In the next step, dead cells were excluded based on the life/dead staining. CD45+ cells were then gated and finally lymphocytes were gated based on their CD45 expression and granularity (SSC). Subsequently, CD56+CD3- NK cells as well as CD3+CD56+/- T cells were defined. NK cells were further discriminated into either donor or recipient origin based on HLA-A2 expression. In this example, the donor is HLA-A2+ and donor NK cells (CD56+HLA-A2+) are displayed in pink, whereas the recipient is HLA-A2- and recipient NK cells (CD56+HLA-A2-) are shown in purple. T cells were further divided into CD3+CD4+ T cells and CD3+non-CD4+ (“non-CD4+”) T cells. Finally, donor as well as recipient origin for both CD4+ and non-CD4+ T cells was determined based on HLA-A2 expression (donor T cells HLA-A2+, recipient T cells HLA-A2-) and are displayed in different blue shades. (B) High proportions of donor NK and T cells can be detected in recipient blood directly post DLTx. Frequencies of donor NK and T cell subsets in peripheral blood of DLTx recipients were analyzed pre, directly post (T0), 24 hours (T24) and three weeks after transplantation (3wk). Donor and recipient cells were discriminated via HLA mismatch using anti-HLA-A2 specific Ab. Gating strategy is shown in Figure 1C . Statistical analysis: one-way ANOVA with Dunn´s multiple comparison test, (n = 39). Data are shown as mean ± SEM, asterisks indicate p-values with ****p < 0.0001. [file Image_1.tiff]

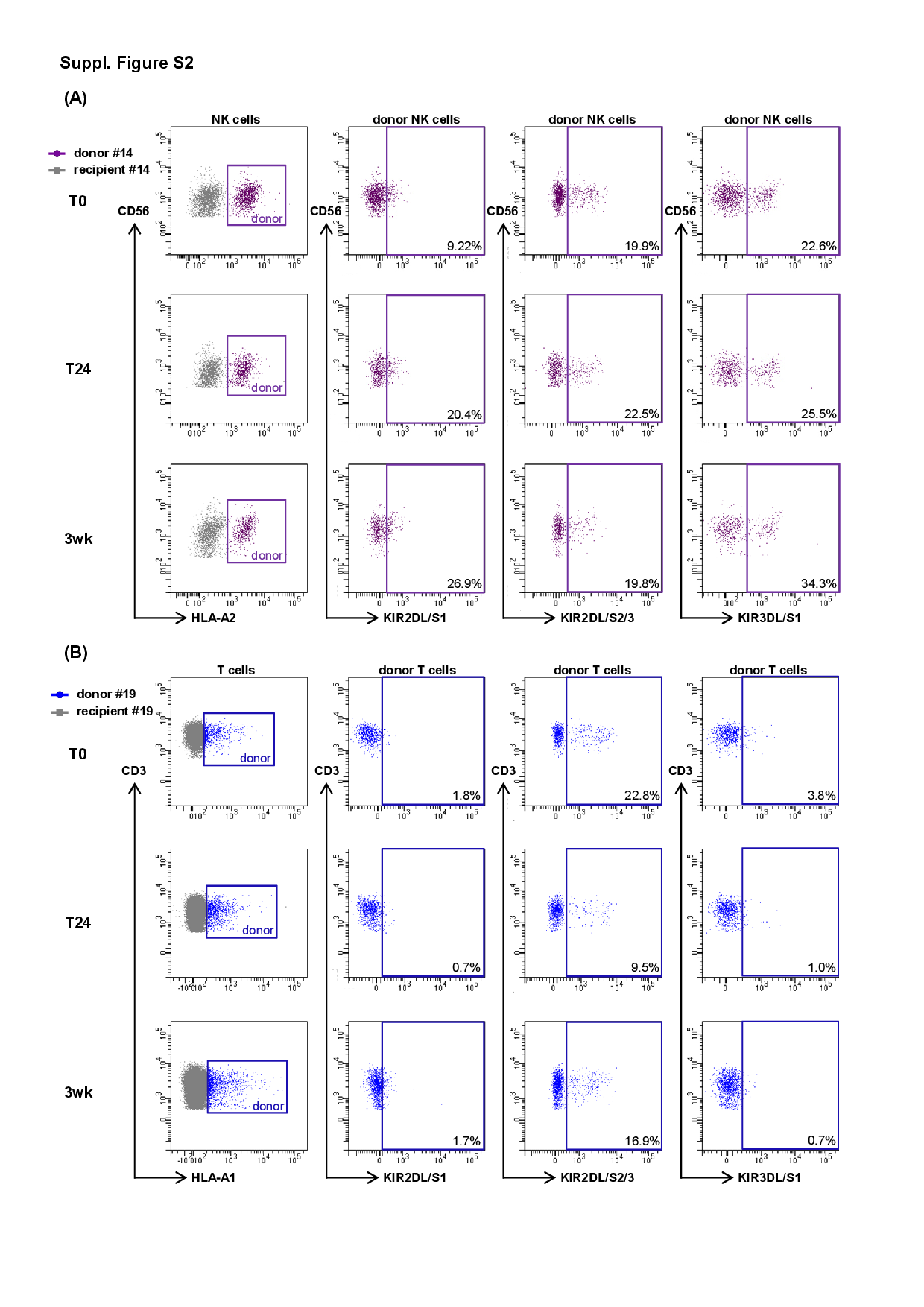

Supplement: Supplementary Figure 2 — Exemplary flow cytometric analyses for KIR2DL/S1, KIR2DL/S2/3 and KIR3DL/S1 surface expression on donor NK and T cells in peripheral blood of double-lung transplant recipients directly post (T0), 24 hours (T24) and three weeks after transplantation (3wk). Donor and recipient cells were distinguished by HLA mismatch: NK cells of the representative donor #14 were identified by HLA-A2 staining (A); T cells of the representative donor #19 were identified by HLA-A1 staining (B), the gating strategies are shown in Figure 1C and S1 . (A) Representative FACS plots of one patient of donor#14 (HLA-A2+; purple) and recipient#14 (HLA-A2-; grey) KIR on NK cells directly after DLTx (T0), 24 hours (T24) and 3 weeks following DLTx. (B) Representative FACS plots of one patient of donor#19 (HLA-A1+; blue) and recipient#19 (HLA-A1-; grey) KIR on T cells. [file Image_2.tiff]

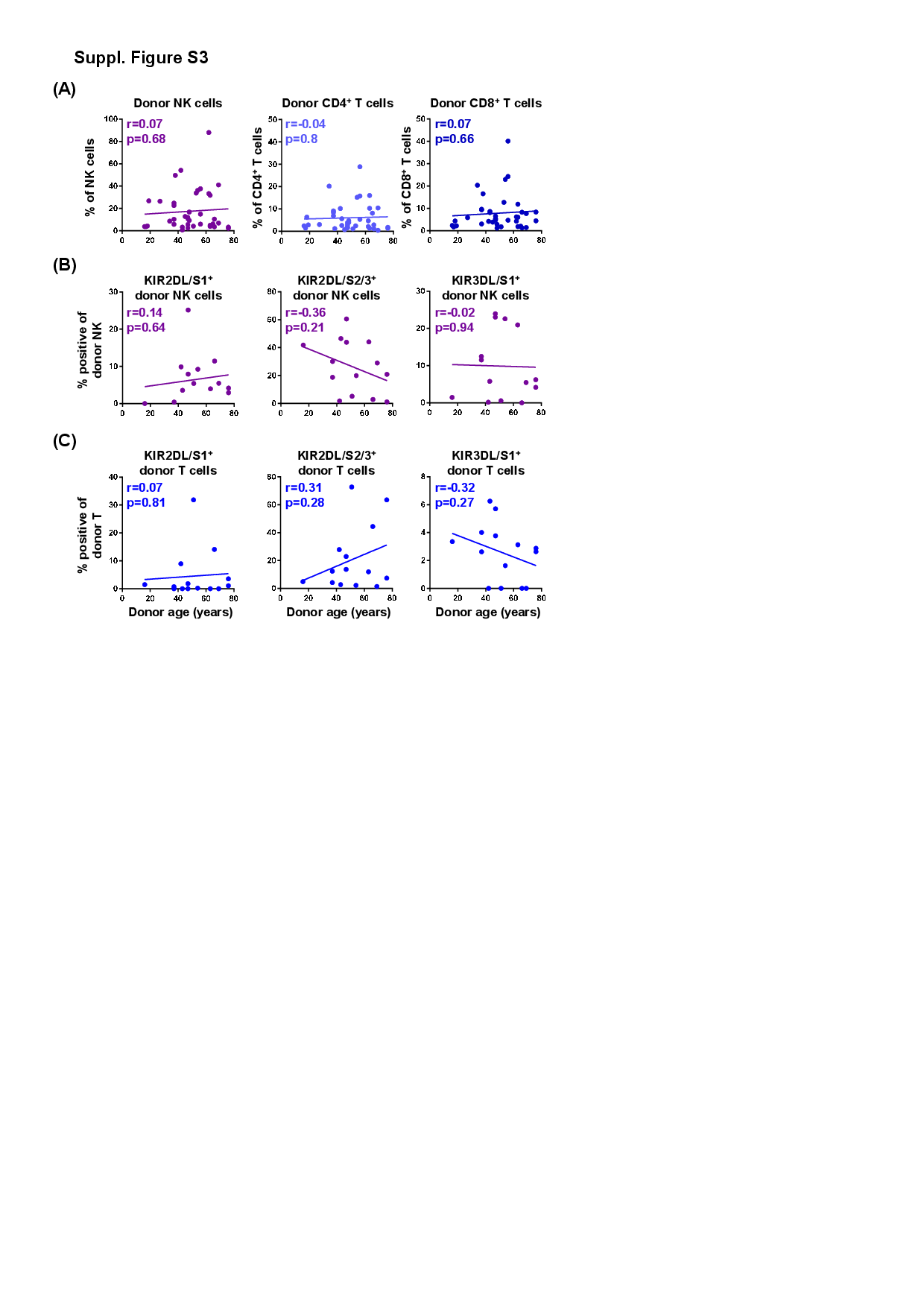

Supplement: Supplementary Figure 3 — Donor age might have no impact on the KIR repertoire on donor NK and T cells directly post DLTx (T0). Linear regression of donor age to (A) donor NK and T cell subsets (n = 39) directly post DLTx (T0) and to KIR on (B) donor NK cells (n = 14) and (C) donor T cells (n = 14) directly post DLTx (T0) is shown. Each dot represents one patient. Gating strategy for donor NK and T cells is shown in Figure 1C and for KIR in Figures 2A, E . Statistical analysis: linear regression and correlation analysis by Pearson correlation. [file Image_3.tiff]

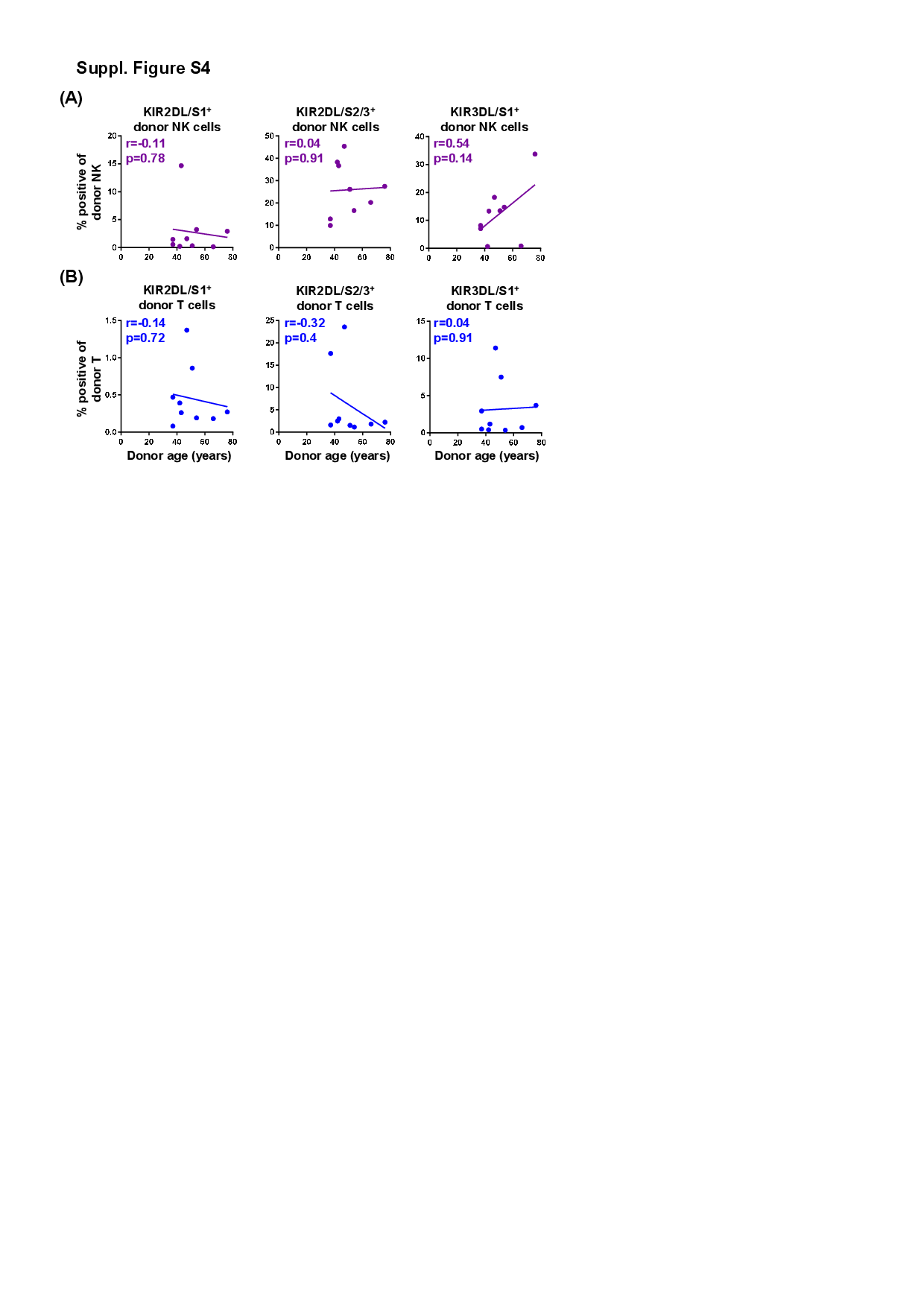

Supplement: Supplementary Figure 4 — Donor age may has no impact on the KIR repertoire on donor NK and T cells in perfusion solution. Linear regression of donor age to KIR on (A) donor NK cells (n = 9) and (B) donor T cells (n = 9) in perfusion solution is illustrated. Gating strategy for KIR in Figures 2A, E . Each dot represents one patient. Statistical analysis: linear regression and correlation analysis by Pearson correlation. [file Image_4.tiff]
