# Supplementary material for: Donor NK and T Cells in the Periphery of Lung Transplant Recipients Contain High Frequencies of Killer Cell Immunoglobulin-Like Receptor-Positive Subsets
Source: Front Immunol. 2021 Dec 13;12:778885. doi: 10.3389/fimmu.2021.778885 (PMC8710687; doi:10.3389/fimmu.2021.778885)
Supplement: Supplementary Table 1 — Antibodies for flow cytometry. APC, allophycocyanine; BV, brilliant violet; ECD, energy coupled dye (phycoerythrin-texas red conjugate); FITC, fluorescein isothiocyanate; PB, pacific blue; PE, phycoerythrin; PerCP, peridinin-chlorophyll-protein complex. [file Table_1.docx]

| **Table S1. Antibodies for flow cytometry** | | | |
| --- | --- | --- | --- |
| **Primary antibodies for HLA staining (all unconjugated)** | | | |
| **Target** | **Isotype** | **Clone** | **Company** |
| HLA-A2, Aw69 | mIgG2b | HB-82 (BB7.2) | Hybridoma supernatant |
| HLA-A2, Aw69 | mIgG1 | HB 117 (PA2.1) | Hybridoma supernatant |
| HLA-A3 | mIgM | Bulk monoclonal | OneLambda |
| HLA-A1, A11, A26 | mIgM | Bulk monoclonal | OneLambda |
| HLA-A23, A24 | mIgM | Bulk monoclonal | OneLambda |
| **Secondary antibodies for HLA staining** | | | |
| **Target** | **Fluorochrome** | **Clone** | **Company** |
| Goat anti-mouse IgG (H+L) | PB | polyclonal | Thermo Fisher |
| F(ab’)_2_ Fragment goat anti-mouse IgG + IgM (H+L) | PE | polyclonal | Dianova (Jackson Immuno Research) |
| Goat anti-mouse IgG + IgM (H+L) | FITC | polyclonal | Dianova (Jackson Immuno Research) |
| **Antibodies for T and NK cell staining** | | | |
| **Target** | **Fluorochrome** | **Clone** | **Company** |
| CD3 | PerCP | SK7 | BD Biosciences |
| CD4 | PE | RPA-T4 | BD Biosciences |
| CD4 | PerCP | SK3 | BD Biosciences |
| CD4 | FITC | RPA-T4 | BD Biosciences |
| CD4 | APC | 13B8.2 | Beckman Coulter |
| CD8 | Pacific Blue | RPA-T8 | BD Biosciences |
| CD16 | APC | B73.1 | BD Biosciences |
| CD16 | BV605/Qdot605 | 3G8 | Biolegend |
| CD16 | FITC | 3G8 | Beckman Coulter |
| CD16 | Pacific Blue | 3G8 | BD Biosciences |
| CD25 | FITC | B1.49.9 | Beckman Coulter |
| CD45 | Alexa Fluor 700 | HI30 | Biolegend |
| CD45 | APC-H7 | 2D1 | BD Biosciences |
| CD45 | AmCyan | 2D1 | BD Biosciences |
| CD56 | APC | N901 | Beckman Coulter |
| CD56 | PE | B159 | BD Biosciences |
| CD56 | ECD | N901 | Beckman Coulter |
| CD69 | PE | TP1.55.3 | Beckman Coulter |
| CD107a | FITC | H4A3 | BD Biosciences |
| KIR2DL/S1/p58.1/  CD158a | FITC | HP-3E4 | BD Biosciences |
| KIR2DL/S2/3/p58.2/  CD158b1/b2 | PE | GL183 | Beckman Coulter |
| KIR3DL/S1/  CD158e | ECD | Z27.3.7 | Beckman Coulter |
|  |  |  |  |
| **Antibodies for isotype control** | | | |
| **Isotype** | **Fluorochrome** | **Clone** | **Company** |
| Mouse IgG1, κ | unconjugated | MOPC-21 | Sigma-Aldrich |
| Mouse IgG2a, κ | unconjugated | UPC-10 | Sigma-Aldrich |
| Mouse IgM | unconjugated | MOPC-104E | Sigma-Aldrich |
| IgG2a | FITC | 7T4-1F5 | Beckman Coulter |
| IgG2a | PerCP | X39 | BD Biosciences |
| mIgG2a | APC | X39 | BD Biosciences |
| mIgG2a | Pacific Blue | G155-178 | BD Biosciences |
| IgG1 | ECD | 679.1Mc7 | Beckman Coulter |
|  |  |  |  |
| APC, allophycocyanine; BV, brilliant violet; ECD, energy coupled dye (phycoerythrin-texas red conjugate); FITC, fluorescein isothiocyanate; PB, pacific blue; PE, phycoerythrin; PerCP, peridinin-chlorophyll-protein complex; | | | |
